# Increased Anion Exchanger-1 (Band 3) on the Red Blood Cell Membrane Accelerates Scavenging of Nitric Oxide Metabolites and Predisposes Hypertension Risks

**DOI:** 10.1093/function/zqae052

**Published:** 2024-12-04

**Authors:** Li-Yang Chen, Pin-Lung Chen, Si-Tse Jiang, Hui-Lin Lee, Yen-Yu Liu, Alysa Chueh, Jing-Heng Lin, Caleb G Chen, Chung-Lieh Hung, Kate Hsu

**Affiliations:** The Laboratory of Immunogenetics, Department of Medical Research, MacKay Memorial Hospital, Tamsui, New Taipei City 251020, Taiwan; The Laboratory of Immunogenetics, Department of Medical Research, MacKay Memorial Hospital, Tamsui, New Taipei City 251020, Taiwan; National Laboratory Animal Center, National Applied Research Laboratories, Taipei 106214, Taiwan; The Laboratory of Immunogenetics, Department of Medical Research, MacKay Memorial Hospital, Tamsui, New Taipei City 251020, Taiwan; Department of Critical Care Medicine, MacKay Memorial Hospital, Tamsui, New Taipei City 251020, Taiwan; Institute of Biomedical Sciences, MacKay Medical College, New Taipei City 252005, Taiwan; Division of Cardiology, Department of Internal Medicine, MacKay Memorial Hospital, Taipei 104217, Taiwan; The Laboratory of Immunogenetics, Department of Medical Research, MacKay Memorial Hospital, Tamsui, New Taipei City 251020, Taiwan; The Laboratory of Immunogenetics, Department of Medical Research, MacKay Memorial Hospital, Tamsui, New Taipei City 251020, Taiwan; Department of Hematology, MacKay Memorial Hospital, Taipei 104217, Taiwan; Department of Hematology, GCRC Laboratory, Mackay Memorial Hospital, New Taipei City 251020, Taiwan; Institute of Molecular Medicine, National Tsing-Hua University, Hsin-Chu 300044, Taiwan; MacKay Junior College of Medicine, Nursing, and Management, New Taipei City 252005, Taiwan; Institute of Biomedical Sciences, MacKay Medical College, New Taipei City 252005, Taiwan; Division of Cardiology, Department of Internal Medicine, MacKay Memorial Hospital, Taipei 104217, Taiwan; The Laboratory of Immunogenetics, Department of Medical Research, MacKay Memorial Hospital, Tamsui, New Taipei City 251020, Taiwan; Institute of Biomedical Sciences, MacKay Medical College, New Taipei City 252005, Taiwan; MacKay Junior College of Medicine, Nursing, and Management, New Taipei City 252005, Taiwan

**Keywords:** red blood cell, nitric oxide, nitrite, blood pressure, anion exchanger-1 (AE1, band 3), anion transport, glycophorin, GP.Mur (Mi.III), Miltenberger blood type, hypertension

## Abstract

The erythrocyte membrane is highly specialized with ∼1 million anion exchanger-1 (AE1) per cell for rapid membrane permeation of HCO_3_^−^_(aq)_, as most blood CO_2(g)_ is carried in this hydrated anionic form. People with the GP.Mur blood type have more AE1 on their erythrocyte membrane, and they excrete CO_2(g)_ more efficiently. Unexpectedly, GP.Mur/increased AE1 is also associated with higher blood pressure (BP). To solve this, we knocked the human *GYP.Mur* gene into C57BL/6J mice at 3′-UTR of *GYPA* to generate GPMur knock-in (KI) mice. KI of human *GYP.Mur* increased murine AE1 expression on the red blood cells (RBC). GPMur KI mice were naturally hypertensive, with normal kidney functions and lipid profiles. Blood NO_3_^−^ [the stable nitric oxide (NO) reservoir] was significantly lower in the GPMur mice. GPMur KI also accelerated AE1-mediated NO_2_^−^ influx into the RBCs and intraerythrocytic NO_2_^−^/NO processing. From tests with different categories of antihypertensives, hypertension in GPMur mice responded best to direct arterial vasodilator hydralazine, suggesting that vasodilator deficiency is the leading cause of “GPMur/AE1-triggered hypertension.” In conclusion, we showed that GPMur/increased AE1 predisposed hypertension risks. Mechanistically, higher AE1 expression increased RBC membrane permeability for NO_2_^−^ and consequently accelerated erythroid NO_2_^−^/NO metabolism; this is associated with lower NO bioavailability and higher BP. As hypertension affects a quarter of the world population and GP.Mur is a common Southeast Asian (SEA) blood type, this work may serve as a primer for “GPMur (biomarker)-based” therapeutic development for hypertension.

## Graphical Abstract

**Graphical Abstract:**
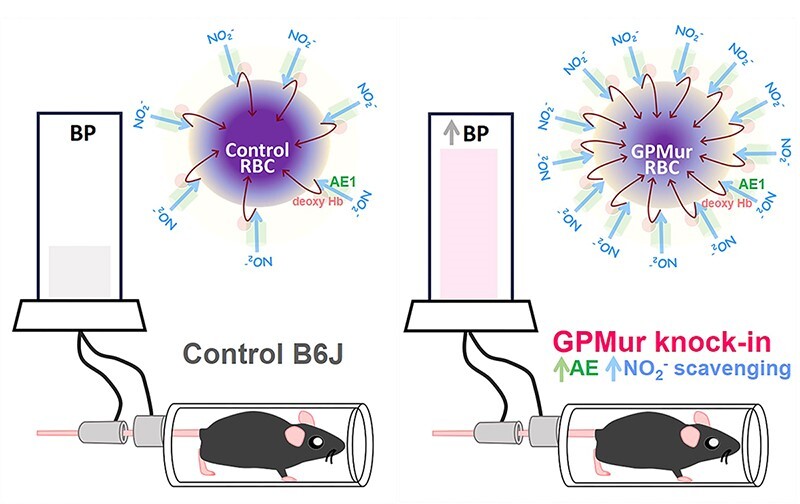

## Introduction

Red blood cell (RBC)-enclosed hemoglobin (Hb) is the main nitric oxide (NO) scavenger in the bloodstream. It has been known for decades that the anion exchanger-1 (AE1) anion transporter (commonly known as band 3) on the RBC membrane partakes in erythroid processing of NO metabolites^1–3^ and essential hypertension,^4^ but how AE1 is involved remains unclear. Anion exchanger-1 transports monoanions (eg, Cl^−^, HCO_3_^−^, NO_2_^−^, and NO_3_^−^) across the cell membrane, and the direction of the transport primarily follows the concentration gradients of individual monoanion species.^5,6^ In respiratory physiology, AE1 passes one HCO_3_^−^ anion in exchange of a Cl^−^ anion (a common counterion for electroneutrality) from the other side of the erythroid membrane in an antiport fashion. Most CO_2(g)_ in the vasculature is transported in the form of HCO_3_^−^_(aq)_.^6–9^ HCO_3_^−^ permeation in and out of the RBCs synchronizes with intraerythrocytic conversion CO_2(g)_ ⇌ HCO_3_^−^_(aq)_, an extremely rapid reaction catalyzed by carbonic anhydrase II (CAII)- mostly present inside the RBCs. HCO_3_^−^ transport via AE1 is the rate-limiting step for this conversion reaction because (1) nonpolar CO_2_ is permeable to the cell membrane but HCO_3_^−^ is not and (2) HCO_3_^−^ transport via AE1 is approximately one order slower than the rate of CAII enzymatic activities.^10,11^

The GP.Mur blood type (also known as the Miltenberger Mi.III subtype) belongs to the MNS system, one of the most complex human blood group systems. The GP.Mur phenotype is present in 2%-23% of Southeast Asians (SEA),^12–17^ and is accompanied by higher AE1 protein expression.^18^ The protein entity of the GP.Mur blood type, or GPMur, is a glycophorin B-A-B hybrid that evolved from homologous gene recombination between *glycophorin B (GYPB)* and *glycophorin A (GYPA)*.^19^ Glycophorin A (GPA) and AE1 are the two most abundant membrane proteins on the RBCs. *GYPB* is a duplicate gene of *GYPA*. While *GYPA* is encoded in both human and mouse genomes, *GYPB* and *GYPB*-derived hybrid genes (eg, *GYP.Mur*) are only encoded in human, and not mouse. Both glycophorin B (GPB) and GPA are erythrocyte-specific and function as membrane protein chaperones—GPB promotes Rh/RhAG complex expression^20^ and GPA binds AE1 and facilitates folding and complex formation of AE1.^21^

People with the GP.Mur blood type express more AE1 because GPMur contains part of the GPA structure that facilitates AE1 expression.^18,22–24^ Higher AE1 expression increases HCO_3_^−^ permeation across the RBC membrane, thus accelerating intraerythrocytic CO_2_/HCO_3_^−^ conversion and the rate of blood CO_2_ transport and respiration. This is demonstrated in two human respiratory-exercise studies that compared subjects with and without the GP.Mur blood type.^25,26^

Intriguingly, healthy people with GP.Mur have slightly higher blood pressure and smaller NO-dependent vasodilatory responses.^27,28^ The incidence of early-onset hypertension also appears higher among Taiwanese carrying the GP.Mur blood type.^29^ Blood pressure and flow-mediated vasodilation (FMD, largely represented by NO-dependent vasodilation) are both more sensitive to individual Hb levels in people with GP.Mur, compared to those lacking the blood type.^27–29^ To verify whether the association between GP.Mur and higher blood pressure (BP) could be directly related or could just be part of a complex association, here we created a GPMur knock-in (KI) mouse model. In it, human GPMur increased murine AE1 expression on the RBC membrane, triggered hypertension, and lowered blood NO_3_^−^ contents. Since AE1 transports NO_2_^−^ and NO_3_^−^ (the main NO metabolites),^5,30^ we examined the influence of GPMur/increased AE1 on RBC processing of NO_2_^−^/NO and on blood pressure responses to different categories of antihypertensives. From the distinct responses, this hypertensive mouse model reveals a novel mechanism associated with the GPMur/increased AE1 phenotype; we thus named it “GPMur/AE1-triggered hypertension.”

## Methods

This Miltenberger KI mouse study was approved by the Institutional Animal Care and Use Committee (IACUC) of Taiwan MacKay Memorial Hospital (MMH IACUC registration: 111-35).

### Generation of Human GPMur KI Mice

**Figure 1.**
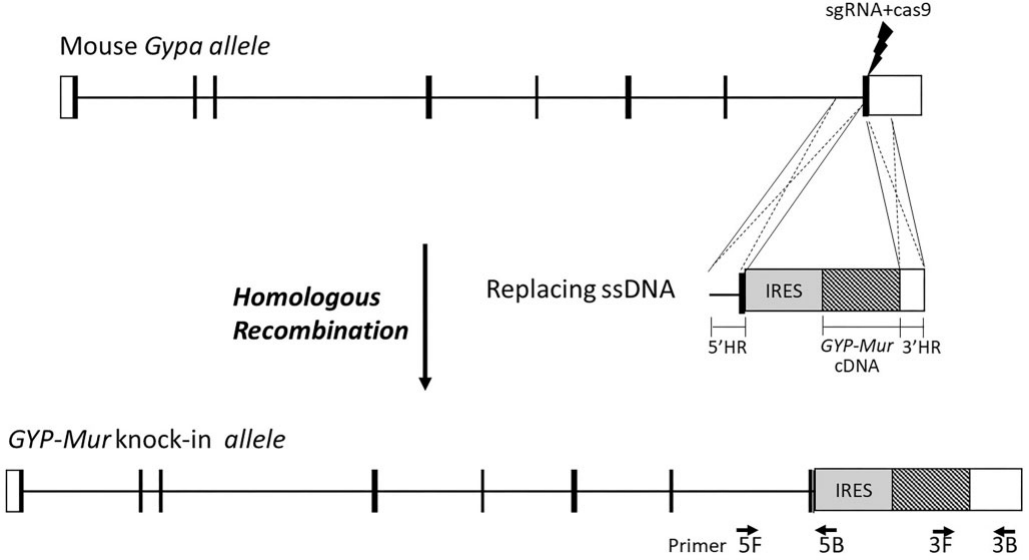
Design of the human GPMur knock-in mouse (GPMur KI) model by CRISPR/Cas9-mediated DNA cleavage and homologous recombination with a replacing ssDNA on the mouse *Gypa allele*. The cleavage site for the designed CRISPR/Cas9-mediated recombination was located after the stop codon of mouse *Gypa*. The target regions of the primer pairs for PCR genotyping are indicated. Abbreviations: 3′HR, 3′ homologous arm; 5′HR, 5′ homologous arm; IRES, polio internal ribosome entry site.

C57BL/6JNarl-*Gypa^eml(GYPMur)MMH^* was generated by CRISPR/Cas9-mediated DNA cleavage and homologous recombination with a replacing ssDNA on mouse *Gypa allele* ^31^ (Figure 1). The targeting construct pUC57-GypaHR-IRES-GYP-Mur contains a polio internal ribosome entry site (IRES) and human *GYP.Mur* cDNA (accession EU338225) flanked by 5′- and 3′-homologous arms . Mouse zygotes were obtained by mating superovulated C57BL/6J females and males (National Laboratory Animal Center, Taiwan). To find out whether KI was successful, mouse genomic DNA was extracted from the tail tips of the pups for polymerase chain reaction (PCR)-sequencing. To reduce deleterious off-target effects and mosaicism from CRISPR/Cas 9-induced gene recombination,^32,33^ GPMur KI founders (F_0_) were crossed with C57BL/6J mice for 3 generations. We then intercrossed hemizygotic GPMur KI mice to obtain the first homozygous GPMur KI mice, and produced stable homozygous progenies by intercrossing homozygous GPMur KI mice. C57BL/6J mice (control) were purchased from the Taiwan National Laboratory Animal Center and were used as the control mice in this study.

### Reverse transcription (RT)-PCR for *GYP.Mur* Transcript in Murine Peripheral Blood

Due to the scarcity of the mouse blood samples, peripheral blood samples were pooled from 6 mice per group, followed by total RNA extraction using the AllPrep^®^ DNA/RNA Micro Kit (QIAGEN, Hilden, GERMANY). Reverse transcription used HiScript II Q RT SuperMix for qPCR (Vazyme, Nanjing, PRC), followed by PCR (i-MAX II PCR PreMix Kit, iNtRON’s Maxime, Seongnamsi, Korea). The forward and reverse primers for *GYP.Mur* cDNA PCR were GBexon2-3nt90F (5′-CAGACAAATGATAAGCAC-3′), which comprises the ending sequence of exon 2 and the beginning sequence of exon 3, and GB453r (5′-GGCATAAGCAAAGGAATAGCAGG-3′). The forward and reverse primers for *GAPDH* cDNA PCR were mouse-GAPDH-F (5′-GTTGTCTCCTGCGACTTCA-3′) and mouse-GAPDH-R (5′-GGAAGATGGTGATGGGATT-3′).

### Complete Blood Count and Serum Biochemical Tests

For complete blood count (CBC), ∼100 μL of whole blood was collected from the facial vein of a mouse using a lancet.^34^ CBC was performed on whole blood samples in EDTA-containing microtubes (BD Vacutainer K2EDTA) by the ProCyte Dx Hematology Analyzer (IDEXX, Westbrook, Maine, USA) at the Taiwan Mouse Clinic.

To evaluate renal functions and serum lipids, at least 100 μL of whole blood per mouse was collected into a serum separation tube (BD Vacutainer SST II), followed by 10 min of 1500 × *g* centrifugation for the serum fraction.^35^ Serum creatinine (CRE), blood urea nitrogen (BUN), uric acid (UA), triglyceride (TG), and total cholesterol (TCHO) were analyzed using FUJI DRI-CHEM SLIDEs on an automatic clinical chemistry analyzer (FUJIFILM Corporation, Tokyo, Japan).^34,35^

### Immunofluorescence Imaging

Murine RBCs were washed and then fixed in 1% paraformaldehyde (PFA)-containing PBS for 10 min at 4°C. The murine RBCs were then immunolabeled with TER-119 (BioLegend, San Diego, USA), which likely targets a sialyl epitope associated with murine glycophorin.^36^ Secondary labeling for TER-119 utilized Alexa Fluor 488-conjugated anti-rat Fab fragment antibody. Confocal images for the immunolabeled RBCs were captured using Leica TCS SP8 X (Leica Microsystems, Wetzlar, Germany). For quantitative assessment, the fluorescence intensities of 20 RBCs from each confocal image were computed and compared by a 2-sample *t-*test.

### Flow Cytometry

Peripheral blood was withdrawn from 2 generations of the GPMur mice at 5 and 8 months old and from age-matched control mice. The RBC fraction was washed with PBS, followed by indirect immunostaining with PJ90929 (custom-made rabbit anti-GPMur against the epitope YPPEEETGETGQ-C located in the extracellular and non-glycosylated region of GPMur) and then with Alexa Fluor 488-anti-rabbit conjugate. The isotype controls were RBC samples stained only with Alexa Fluor 488-anti-rabbit conjugate (the secondary Ab) and not with PJ90929 (the primary Ab). Flow cytometric data were collected and analyzed using FACSCalibur (BD).

### AE1 Western Blot

The RBC membrane fractions (ghosts) were extracted by hypotonic rupture and then solubilized in an equal volume of the doubly concentrated lysis buffer containing 2% CHAPS (3-[(3-cholamidopropyl)dimethylammonio]-1-propanesulfonate), 2% Nonidet *P*-40, 0.05% SDS, 2× PBS, and Roche’s cOmplete^TM^ protease inhibitor cocktails (Sigma-Aldrich, MA, USA). After rigorous protein lysis with multiple freeze-and-thaw steps, lysate concentrations were determined by the Lowry assay (DC Protein Assay Kit; Bio-Rad, CA, USA), as described.^18^ Protein samples were further denatured in the Lithium dodecyl sulfate (LDS) sample buffer supplemented with 10% β-mercaptoethanol at 70°C for 30 min prior to sodium dodecyl sulfate polyacrylamide gel electrophoresis (SDS-PAGE). For each sample, 20 μg of the lysate was loaded per well on a 4%-12% gradient Bis-Tris gel (Thermo Fisher Scientific, CA, USA). Protein signals were developed using the Super Signal West Pico PLUS Chemiluminescent Substrate (Thermo Fisher Scientific). Murine AE1 was identified using a combination of 3 anti-AE1 antibodies (BRIC 132, BRIC 155, and mBRIC 6). Both BRIC 132 and BRIC 155 target regions in the intracellular C-terminal domain of AE1, which is identical in human and mouse (International Blood Group Reference Laboratory, Bristol, UK).^37^ The epitope of the custom-made “mBRIC 6” rabbit polyclonal Ab is an extracellular of mouse AE1 that corresponds to the epitope sequence of human BRIC 6 mAb (murine AE1 protein sequence 564-585: QDYPLQQTYAPVVMKPKPQGPV).^38^

### Murine AE1 enzyme-linked immunosorbent assay (ELISA)

For each adult mouse over 20 g, 50-100 μL of blood was withdrawn for protein analyses. RBC ghosts were isolated by hypotonic rupture and then centrifugal fractionation at 2-4°C. The RBC membrane fraction of each mouse was solubilized in an equal volume of the doubly concentrated, ice-cold lysis buffer (the final composition of the ghosts after mixing with an equal volume of the 2× lysis buffer: 1% NP-40 in PBS supplemented with Roche’s cOmplete^TM^ protease inhibitor cocktails). Membrane protein lysis included multiple rounds of “freeze-and-thaw” and rotator mixing with intermittent vortex at 2-4°C. Protein concentrations of the lysates were determined by the Lowry method. An ELISA plate was coated with BRIC 155 at 4°C overnight. After overnight coating, 2 μg of each mouse lysate sample was added to a well of the BRIC 155-coated plate for a 2-h incubation at 37°C, followed by PBS washes. Bound AE1 on the ELISA plate was then reacted with the detecting Ab mBRIC 6 (1:50 000 ∼ 1:100 000 dilution) for 1.5 h at 37°C, followed by PBS washes. Anion exchanger-1-mBRIC 6 interaction was magnified by Horseradish peroxidase (HRP)-conjugated anti-rabbit IgG (1:3000 dilution) for 30 min at 37°C, followed by HRP-based quantification.

For quantitative calibration in this ELISA, the peptide used to immunize the rabbits to make mBRIC 6 Ab (AA 564-585: QDYPLQQTYAPVVMKPKPQGPV) was serially diluted and then coated onto the same ELISA plate overnight at 4°C. The wells coated with different concentrations of the mBRIC 6 peptide were incubated with mBRIC 6 Ab and then with HRP-conjugated anti-rabbit IgG, concurrently with the above reaction using the lysate samples .

### Time-lapse Imaging for DAF-FM-loaded RBCs

The protocol was described previously for human RBC experiments,^30^ with slight modification for the mouse experiments here. One-day-old refrigerated whole blood samples from GPMur KI and the control mice were washed and incubated in 6 μm DAF-FM at 37°C for 20 min, followed by washes with PBS and then with HBSS. These DAF-FM-loaded RBCs (1% in HBSS) were put in a slide chamber (μ-Slide VI-Flat, ibidi, Gräfelfing, Germany). The NO/N_2_O_3_-sensitive fluorophore DAF-FM was excited at 488 nm, and its emission at 510-550 nm was recorded every 2 s for 20 min using the Leica TCS SP8 X Confocal Spectral Microscope Imaging System. In the beginning of the recording, NaNO_2_ (final 1 mm) was added to a slide chamber containing 20 μL of DAF-FM-loaded RBCs. With this experimental setting, DAF-FM fluorescence intensities inside the RBCs generally increased and plateaued in 10-20 min after the addition of nitrite. To analyze the time-lapse recording, 20 DAF-FM-loaded RBCs from the recording of each mouse were selected. The fluorescence intensities were normalized (0%-100%) for kinetic analyses. The time-lapse data were fitted using a logistic function to estimate the duration needed to reach 50% of the maximal DAF-FM fluorescence intensities.

### Measurements of Blood NO Metabolites

The plasma fractions isolated from murine whole blood samples were filtered using NANOSEP 3 K OMEGA ultrafilters (Pall Lab, NY, USA) by centrifugation (14 000 × *g*) at 4°C for 10-20 min. The filtrates (10 μL each) were analyzed using the Nitrate/Nitrite assay kit (Cayman Chemical, MI, USA).

### Murine BP Measurements

Mouse BP was measured using an 8-channel CODA High Throughput Non-invasive Blood Pressure System (Kent Scientific Corp., Torrington, CT, USA), a widely used instrument for murine BP (reported up to 99% correlation to the invasive telemetry approach).^39,40^ The CODA BP system is a traditional, tail-cuff method, with a volume-pressure recording (VPR) sensor-cuff (similar to a stethoscope) and an occlusion cuff (similar to the arm cuff for conventional human BP measurement). The instrument settings in this study were 5 acclimation cycles, 15 cycles per set, 250 mmHg as the maximal occlusion pressure, and 20 s for deflation.^41^ Familiarization and training are required for the animals to be comfortable with the tail-cuff procedure. In a typical session, the experimenter gently placed a mouse in a CODA holder or at the entry point of the holder, from which the trained mouse could voluntarily walk into the holder as it was accustomed to the procedure. An occlusion cuff and a VPR cuff were then fitted onto the mouse tail. The holder carrying a cuffed mouse inside was moved to the warming platform and then covered with a thermal blanket; this ensured the temperature of the tail to be within 32-35°C during volume-pressure-based BP measurements. Invalid measurements were mostly due to unmet tail temperature or restless states of the animal, which were identified and excluded following the manufacturer’s guidelines.

### Evaluation of Antihypertensive Medications

Antihypertensives were tested on adult male mice between 4 and 7 months old with body weights (BW) ≥ 25 g. The optimal dose of an antihypertensive drug was determined based on previous publication and our animals’ drug responses.^42,43^ On the morning of a test day, blood pressure and BW of the GPMur KI and age-matched control mice were first measured (for fresh preparation of individual medication). The mice were individually given the medication mixed in 100 μL autoclaved ddH_2_O by oral gavage. Their BP and heart rates (HR) were measured at the 1st, 3rd, and 24th hour post-medication. The BP data collected 24 h later were to assess whether the BP-lowering effects of an antihypertensive drug have been cleared.

The OriginPro program was used for statistics and all graphic preparation. The Dose Response fitting function in OriginPro was used to estimate EC50. Analysis of covariance (ANCOVA) in SPSS (IBM) was used to compare the effect of a drug between the 2 groups of mice. The ANCOVA statistical method considered individual BP before and 1 h after oral gavage of an antihypertensive drug. The 2 groups of mice are distinguished by the absence/presence of GPMur (the between-subjects, fixed factor in ANCOVA). Pre-medicated BP (*t* = 0) was designated as the covariates; BP measured at 1 h post-medication was designated as the dependent variable. By ANCOVA, statistical significance of the fixed factor (expression of GPMur) suggests that the pharmacological responses between the 2 groups could be different.

## Results

### Generation of GPMur KI Mice

GPMur KI mice were generated by inserting human *GYP.Mur* cDNA into the 3′-UTR of murine *GYPA* gene using CRISPR/Cas 9-mediated homologous recombination (Figure 1). All the data presented here were from male homozygous mice from the fifth to the ninth generations. Homozygous GPMur KI pups had 100% postnatal survival and a roughly 1:1 sex ratio, which follows the Mendelian prediction. GPMur KI did not affect the health or lifespan of C57BL/6J mice.

**Figure 2.**
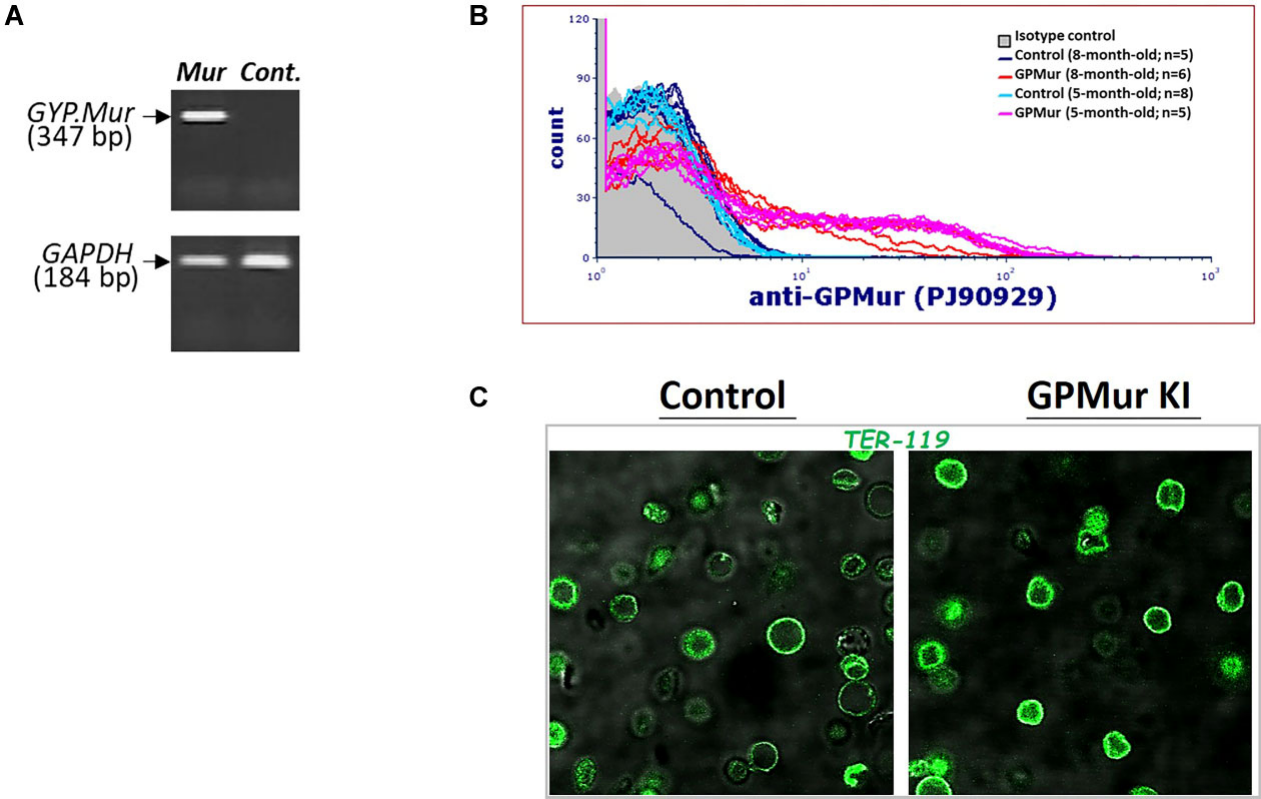
The expression of human GPMur in the KI mice was verified by (A) RT-PCR, (B) flow cytometry, and (C) confocal imaging. (A) RT-PCR revealed the presence of human *GYP.Mur* transcript in the peripheral blood of GPMur KI mice, and not that of the control mice (top). The housekeeping gene *GAPDH* served as the experimental (RT-PCR) control (bottom). (B) GPMur flow cytometry using anti-GPMur Ab (clone PJ90929) revealed the presence of GPMur protein in the RBCs from GPMur KI mice, compared to age-matched control mice. (C) TER-119-stained murine erythrocytes were visualized by confocal microscopy. Scale bar, 10 μm.

The *GYP.Mur* transcript was present in the peripheral blood of GPMur KI mice (Figure 2A). The GPMur protein expression on the RBCs of the KI mice was confirmed by flow cytometry and confocal imaging. For flow cytometry, we labeled RBCs from 2 generations of GPMur KI mice with a GPMur-specific Ab (clone PJ90929), and verified that the GPMur epitope was only present in the KI mice, and not in the age-matched control mice (Figure 2B). We also examined glycophorin expression on the murine RBCs using TER-119, a widely used mAb that likely targets glycophorin-associated sialic acids.^36^ Immunofluorescence staining with TER-119 showed 73.6% ± 53.0% higher fluorescence intensities on the RBCs of GPMur KI mice compared to the RBCs of the control mice (Figure 2C: *P* < 10^−5^), suggesting an increase in the glycophorin-associated sialic acid moiety due to the expression of glycophorin B-A-B hybrid GPMur.

The CBC data of the GPMur mice were all within the normal range of C57BL/6J mice (Supplementary Table 1).^44^ The Hb levels in GPMur KI vs. the control mice were not significantly different. The mean corpuscular Hb concentrations (MCHC) of the GPMur mice were slightly lower than that of the age-matched control mice, but with statistical significance [Supplementary Table 1: 28.5 ± 0.3 g/dL (GPMur KI) vs. 29.2 ± 0.5 g/dL (control); both within the normal range of MCHC for B6J mice ∼30.2 g/L (95% CI ranging 24.6-34.9 g/L)].^44^ For comparison, healthy people with GP.Mur have slightly but significantly lower Hb and MCHC.^45,46^

### Human GPMur Increased Murine Erythroid AE1 Expression

**Figure 3.**
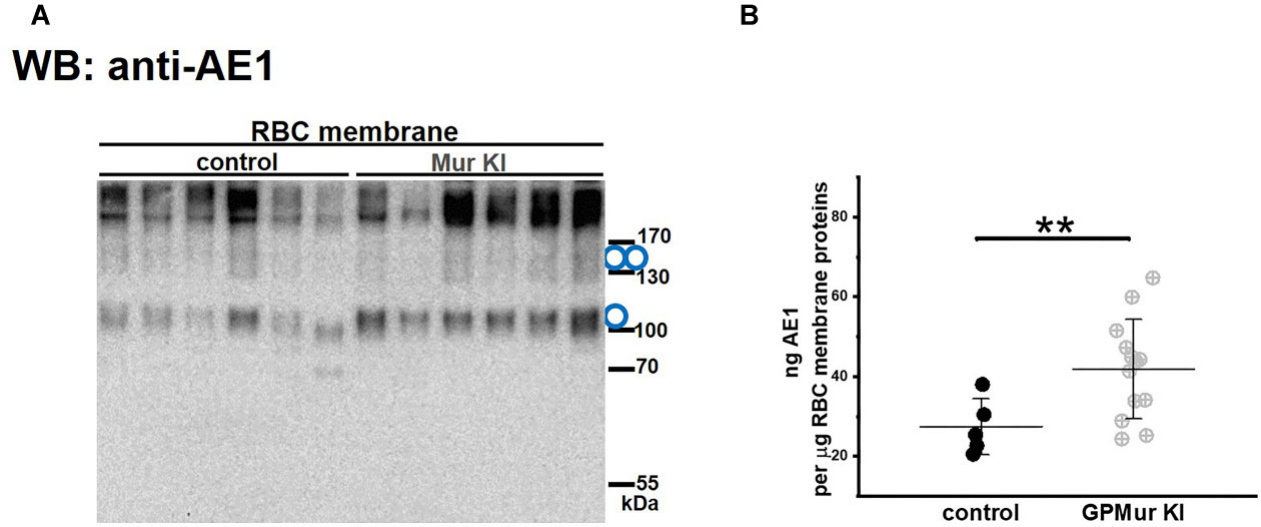
Human GPMur enhanced murine AE1 expression on the RBC membrane. (A) The immunoblot examined AE1 expression levels in the RBC membrane lysates from 6 GPMur KI and 6 control mice, and found more monomeric and dimeric AE1 in the GPMur than the control mice. The circle symbols indicate monomers and dimers of AE1. (B) Mouse AE1 ELISA verified more AE1 present in the erythroid membrane fraction of the GPMur KI than the control mice. Each dot represents the measurement of a mouse. Shown mean ± SD. **P < 0.01 deemed significant by an unpaired *t*-test.

As previously shown in human RBCs and heterologously expressed cultured cells (eg, transfected HEK-293 cells), an important function of GP.Mur is to enhance AE1 expression by stabilizing AE1 folding and complexes on the RBC membrane.^18,22^ Here, by AE1 immunoblot, expression of human GPMur also significantly increased the levels of murine AE1 monomers and dimers on the erythrocyte membrane (Figure 3A).

We also quantitated erythroid AE1 expression by mouse AE1-specific sandwich ELISA. On average, there was 27 ± 7 ng AE1 per μg of the erythroid membrane proteins in the control mice, compared to 42 ± 12 ng AE1 per μg of the erythroid membrane proteins in the GPMur KI mice (Figure 3B). GPMur KI increased murine erythroid AE1 expression by ∼55%. For comparison, by human AE1-specific sandwich ELISA, there is ∼21% more AE1 on the RBC membrane of college elite athletes with the GP.Mur blood type than on the RBC membrane of their peers lacking the phenotype [4.3% ± 2.4% (control) vs. 5.2% ± 1.7% (GPMur+); *P < 0.05].^27^ Thus, KI of human *GYP.Mur* gene into B6J mice increased erythroid AE1 even more than the naturally occurred human GP.Mur RBC type.^25,27^

There are 2 isoforms of AE1—erythroid AE1 (AE1) and kidney AE1 (kAE1), the latter of which is a truncated form of eAE1 that lacks the first 65 amino acids in the N-terminus.^47^ From immunoblot, the kidneys of both GPMur KI and the control mice expressed similarly very low levels of kAE1 (data not shown). This is in agreement with others’ finding that kAE1 expression is not affected by the chaperone activity of erythroid GPA.^48^ This also verifies that murine glycophorin transcripts are exclusively expressed in the erythroid lineage and are absent in other types of tissues, including the kidneys (also confirmed by searches in the Tabula Muris database that contains mouse tissue-specific single-cell transcriptomes^49^).

### GPMur/more AE1 Induced Higher Blood Pressure in Mice

From consecutive 5 generations (3-11 months old), GPMur KI male mice consistently showed significantly higher systolic blood pressure (SBP) and diastolic blood pressure (DBP), compared to age-matched control male mice (Table 1 and Supplementary Figure 1). Their differences in mean arterial pressure (MAP) were 15-27 mmHg at 3-9 months old (equivalent to middle-aged human), and their differences reduced to 7-8 mmHg at 11 month old (corresponding to old age in human^50^) and diminished after 1 year old.

**Table 1.** Blood Pressure of GPMur KI vs. the Control Mice at Different Ages

	3 month old			5 month old			9 month old			11 month old		
GPMur KI male	Control (9)	GPMur (6)	*P*-value	Control (9)	GPMur (7)	*P*-value	Control (8)	GPMur (9)	*P*-value	Control (16)	GPMur (10)	*P*-value
**SBP (mmHg)**	141.8 ± 11.1	164.6 ± 13.1	P* < 0.05	127.4 ± 13.5	143.0 ± 11.0	P* < 0.05	102.7 ± 16.1	128.5 ± 16.0	P* <0.05	99.0 ± 7.0	107.4 ± 10.8	P* < 0.05
**DBP (mmHg)**	109.5 ± 10.9	134.5 ± 12.8	P* < 0.05	93.9 ± 11.7	108.4 ± 10.2	P* < 0.05	73.3 ± 12.3	100.7 ± 16.6	P* <0.05	71.6 ± 5.6	78.0 ± 8.8	P* < 0.05
**MAP (mmHg)**	119.9 ± 10.9	144.2 ± 12.9	P* < 0.05	104.7 ± 12.3	119.6 ± 10.4	P* < 0.05	82.8 ± 13.6	109.6 ± 16.3	P* <0.05	80.2 ± 5.8	87.6 ± 9.5	P* < 0.05
**PP (mmHg)**	32.2 ± 3.6	30.1 ± 1.8	n.s.	33.4 ± 2.1	34.6 ± 1.7	n.s.	29.3 ± 5.0	27.8 ± 2.9	n.s.	27.4 ± 3.5	29.4 ± 3.4	n.s.
**Heart rate (/min)**	521.5 ± 103.2	582.0 ± 42.9	n.s.	480.3 ± 52.7	494.4 ± 53.4	n.s.	517.5 ± 90.9	553.8 ± 63.7	n.s.	466.6 ± 84.4	503.7 ± 76.4	n.s.

Abbreviations: DBP, diastolic blood pressure; MAP, mean arterial pressure; PP, pulse pressure; SBP, systolic blood pressure. *n.s*., statistically not significant (*P* ≥ 0.05).

To probe into the potential factors contributing to “GPMur/AE1-induced hypertension,” we compared their BWs, kidney functions (serum creatinine; BUN; UA) and lipid profiles [total cholesterols (TCHO); Tg]. All their measurements were in the normal range of C57BL/6J mice (compared to the statistics from Charles River Laboratories), and most were not different between GPMur KI and the control mice at young or very old ages (Table 2).^44,51^ Though their serum creatinine levels increased with aging, they were all within the normal range [mean creatinine 0.3 mg/dL (95% CI ranging 0.2-0.5 mg/dL) from the Charles River Laboratories].^44,51^ Their BUN was also normal and comparable, indicating undisturbed renal functions with GPMur KI. Four month old GPMur KI mice even presented significantly lower serum UA than age-matched controls; these differences in serum UA diminished at the very old age. Young GPMur mice had ∼19 mg/dL higher total cholesterols than that the control mice, though their values were all within the normal range of C57BL/6J mice [mean TCHO 114 mg/dL (95% CI ranging 69-169 mg/dL)].^44^ Slightly higher total cholesterols persisted in very old GPMur mice, which were no longer significantly different from the control data (Table 2). On the other hand, as the mice aged, the GPMur mice had significantly lower TGs than the control mice [mean Tg 157 mg/dL (95% CI ranging 67-278 mg/dL)]. In sum, GPMur/AE1-associated hypertension was unlikely due to kidney dysfunction, metabolic, or lipid abnormalities.

**Table 2.** Lipid and Kidney Function Tests for GPMur KI vs. the Control Male Mice at Young (4 Month Old) and Very Old (19 Month Old) Ages

Lab tests	Control at 4 month old (10)	GPMur KI at 4 month old (8)	*P*-value	Control at 19 month old (10)	GPMur KI at 19 month old (8)	*P*-value
**Body weight (g)**	30.4 ± 2.0	28.7 ± 1.7	n.s.	36.5 ± 2.0	34.0 ± 3.1	n.s.
**CRE (mg/dL)**	0.430 ± 0.157	0.338 ± 0.060	n.s.	0.512 ± 0.172	0.503 ± 0.237	n.s.
**BUN (mg/dL)**	27.4 ± 5.1	24.6 ± 1.3	n.s.	28.7 ± 3.6	27.6 ± 3.2	n.s.
**UA-P (mg/dL)**	2.5 ± 0.5	1.8 ± 0.6	*P* < 0.05	3.8 ± 0.8	3.9 ± 0.4	n.s.
**TG (mg/dL)**	96.4 ± 43.8	96.4 ± 43.1	n.s.	176.3 ± 42.0	132.9 ± 39.1	*P* < 0.05
**TCHO (mg/dL)**	109.7 ± 11.3	128.9 ± 8.5	*P* < 0.01	110.2 ± 8.0	120.5 ± 37.5	n.s.

Abbreviations: BUN, blood urea nitrogen; CRE, creatinine; TG, triglyceride; TCHO, total cholesterol; UA-P, uric acid concentration. *n.s*., statistically not significant (*P* ≥ 0.05).

### Lower Systemic NO (Nitrate) Bioavailability in GPMur KI Mice

**Figure 4.**
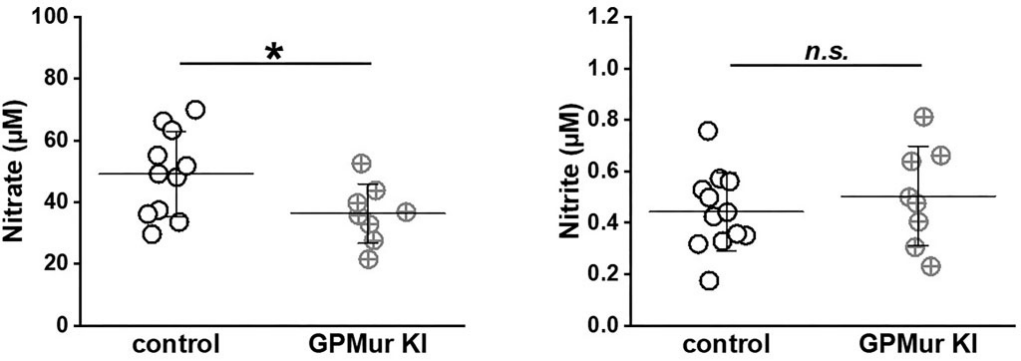
GPMur/more AE1 lowered systemic NO_3_^−^ reservoir. The levels of blood plasma nitrate and nitrite from GPMur KI vs. age-matched control mice were measured. Each dot represents the datum of a mouse measurement. The horizontal bars indicate mean ± SD. *P < 0.05 deemed statistical significance.

Elite college athletes with the GP.Mur blood type present lower FMD and exhale less NO,^27,29^ suggesting NO deficiency associated with the expression of erythroid GPMur/AE1. Here, we compared the blood contents of NO metabolites in these mice. Nitrate (NO_3_^−^, half-life: 5-8 h) is a stable form of NO metabolite in mammalian blood.^52,53^ Nitrite (NO_2_^−^, half-life: 0.5-0.8 h) is a more transient NO metabolite that is actively converted to NO (half-life: < 2 ms) or other NO species mostly by different redox states of Hb inside the RBCs.^52,54–56^ In GPMur KI mice, their blood nitrate levels were significantly lower compared to age-matched control mice [Figure 4: 49.1 ± 13.8 μm (control) vs. 36.3 ± 9.6 μm (GPMur KI)]. On the other hand, their blood nitrite levels were similar. All the mice were housed in the same environment and fed with the same chow; their daily intake of chow was similar (3.6 ± 0.3 g chow consumed per day per GPMur mouse vs. 3.2 ± 0.2 g chow per day per age-matched B6J mouse; n.s. by unpaired *t*-test). Thus, their different plasma nitrate levels were most likely due to innate metabolic differences associated with *GYP.Mur* gene expression.

### GPMur/AE1 Accelerated NO_2_^−^ Influx Into RBCs and Subsequent NO_2_^−^/NO Processing Inside the RBCs

**Figure 5.**
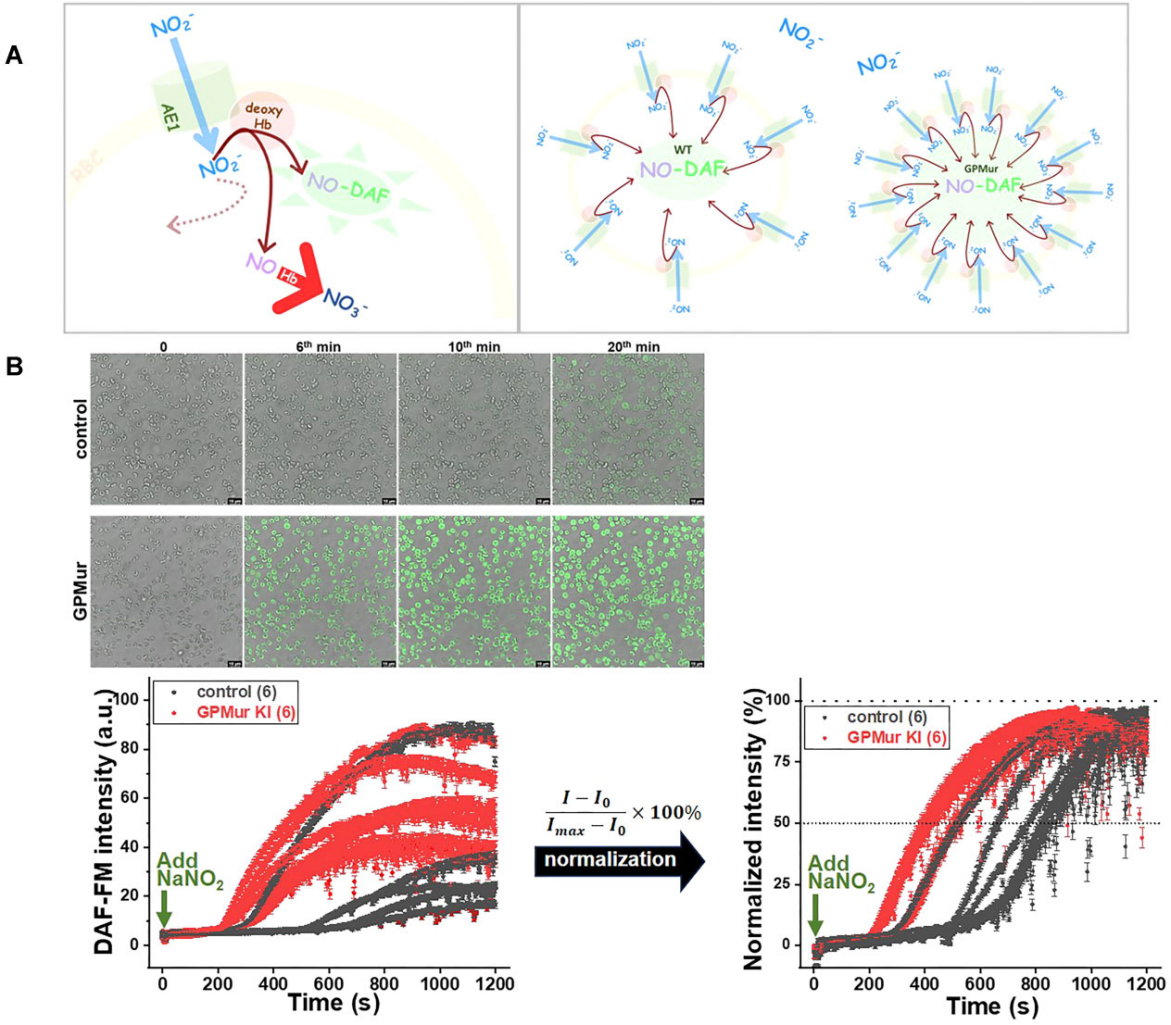
Intracellular DAF-FM labeling of nascent NO production from NO_2_^−^ revealed the influence of GPMur/anion transporter in erythroid NO_2_^−^/NO processing. (A) The experiment rationale: To test whether higher AE1 expression (as in GPMur KI) could influence erythroid NO_2_^−^/NO processing, RBCs were first loaded with NO-sensitive DAF-FM probe, which fluoresces upon binding to NO. Nitrite passes through AE1 to enter RBCs, where it can be converted to NO by deoxyHb. Though nascent NO synthesized from intraerythrocytic nitrite reduction should be scavenged by oxyHb at an extremely fast rate, a fraction of nascent NO is trapped upon binding to DAF-FM, which allows assessment of erythroid NO_2_^−^/NO processing. (B) Top panels show the snapshots of DAF-FM-loaded RBCs from GPMur KI and the control mice right before the addition of nitrite, and at the 6th, 10th, and 20th minute following the nitrite treatment. Left: the fluorescence intensities of 20 DAF-FM-loaded RBCs from each mouse were averaged and plotted in mean ± SEM at each time point. Right: DAF-FM fluorescence intensities were normalized to maximal 100%.

**Figure 6.**
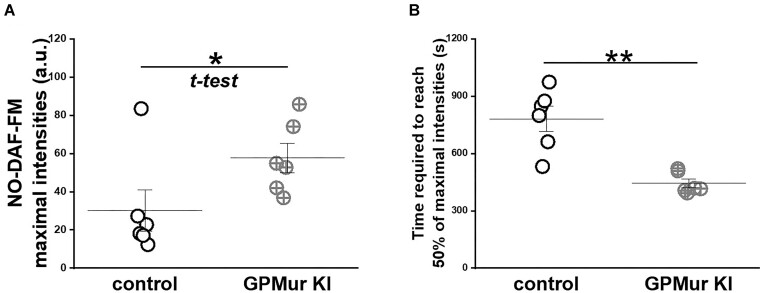
GPMur/more AE1 accelerated and increased erythroid NO_2_^−^/NO metabolism. (A) Before normalization of the DAF-FM fluorescence intensities, RBCs from the GPMur KI mice (*n* = 6) had significantly more nascent NO generated from nitrite reduction, compared to RBCs from the control mice (*n* = 6). (B) After normalization of the DAF-FM intensities, the rates of NO produced from nitrite reduction in the RBCs of the control mice (*n* = 6) were significantly slower, compared to the rate in the RBCs of the GPMur KI mice (*n* = 6). The horizontal bars indicate mean ± SD. **P < 0.01 and *P < 0.05 deemed statistical significance.

Since AE1 transports NO_2_^−^ and NO_3_^−^,^5,57,58^ more AE1 on the GPMur RBCs is expected to increase membrane permeability to these monoanions. To evaluate the impacts of GPMur/increased AE1 on erythroid NO_2_^−^/NO metabolism, we preloaded NO-sensitive fluorophore DAF-FM in the RBCs and then added nitrite into the milieu to stimulate NO_2_^−^ influx. NO_2_^−^ influx into the RBCs drives formation of nascent NO from NO_2_^−^ reduction (NO_2_^−^ → NO) that is catalyzed by deoxyHb. When nascent NO encounters DAF-FM preloaded inside the RBCs, it is trapped in the form of NO-bound DAF-FM, which becomes fluorescent from the non-fluorescent (unbound) form (Figure 5A). By comparing the time course of DAF-FM fluorescence, there was significantly more nascent NO generated from nitrite reduction at significantly faster rates in the GPMur RBCs (Figures 5 and 6). To verify kinetically, DAF-FM fluorescent intensities were normalized to maximal 100% to calculate the time needed to reach 50% of maximal DAF-FM fluorescence after adding NO_2_^−^. GPMur/increased AE1 resulted in 43% faster NO_2_^−^ influx and reduction to nascent NO [the time to reach 50% of the maximal DAF-FM fluorescence in Figure 6: 781±160 s (control) vs. 444 ± 56 s (GPMur)].

Since AE1-facilitated anion transport is estimated to be much slower than the rate of intraerythrocytic NO_2_^−^ reduction to NO,^30,59^ membrane transport of NO_2_^−^ is the rate-limiting step in erythroid NO_2_^−^/NO processing. Thus, we could observe faster erythroid NO_2_^−^/NO processing due to higher AE1 (GPMur) expression (Figure 6). Faster erythroid NO metabolism may use up systemic NO_3_^−^ reservoir more quickly (Figure 4) and drive blood pressure (Table 1). However, the detailed mechanisms or the NO species involved to trigger “GPMur/AE1-associated hypertension” are unknown.

### Differential Antihypertensive Drug Sensitivities

**Figure 7.**
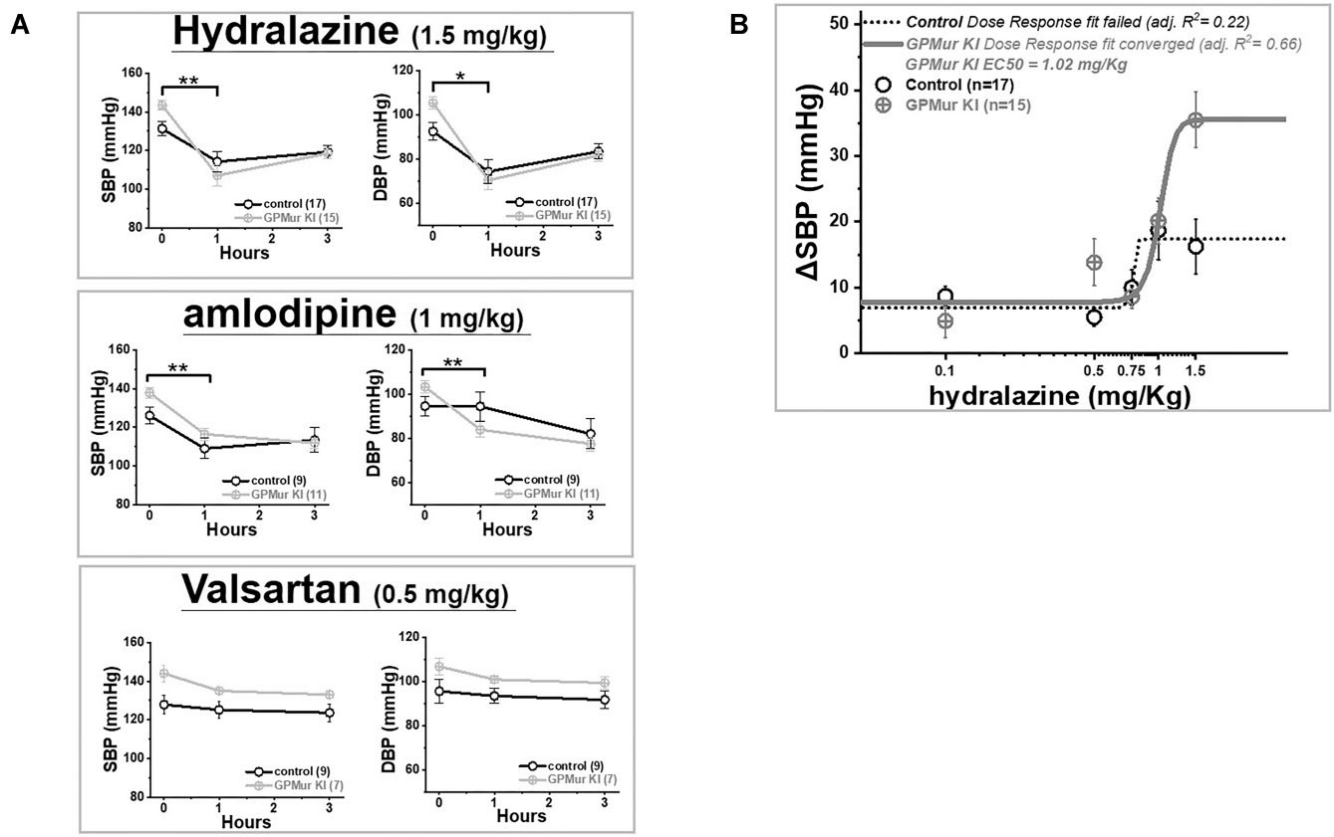
Antihypertensive drug tests revealed GPMur KI as a novel hypertension murine model. (A) Among the popular antihypertensives tested, hydralazine and amlodipine were more effective in BP reduction for GPMur KI (labeled gray) than the age-matched, control mice (labeled black); in contrast, valsartan exerted similar BP-reducing effects. BP at *t* = 0 was measured right before oral gavage of the drug. For each drug tested, the effects of GPMur/AE1 on BP reduction within 10 h post-treatment were assessed by ANCOVA (*P < 0.05 and **P < 0.01 deemed statistically significant). The number of mice per group was indicated in parentheses inside the legend box. Shown mean ± SEM. (B) The dose responses of hydralazine were shown as SBP changes in 1-h hydralazine treatment (∆SBP). Fitting of the dose response curve of hydralazine converged for GPMur KI, but failed for the control mice (shown as a dotted line).

To explore the potential mechanism(s) of “GP.Mur/AE1-associated hypertension,” we tested the mice with different categories of antihypertensive drugs. GPMur KI and the control mice between 4 and 6 months old were tested with dihydropyridine calcium channel blocker (CCB) amlodipine, angiotensin receptor blocker (ARB) valsartan, and direct arterial vasodilator hydralazine. All these antihypertensives lowered BP, but with distinct features (Figure 7 ). Valsartan showed similar BP-lowering effects on GPMur KI and the age-matched control mice, whereas both hydralazine and long-acting amlodipine lowered BP more significantly in GPMur KI than the control mice. Compared to amlodipine, hydralazine exerted even stronger BP-lowering effects on the GPMur KI mice. For verification, we examined the dose response curves of hydralazine on GPMur KI vs. the control mice. Hydralazine showed apparent dose-dependent reduction of SBP in the GPMur mice, with EC50 at 1.02 mg/kg BW (within the range of the recommended doses of hydralazine for human patients). Fitting of the hydralazine dose response curve failed for the control mice, due to insignificant drug effects (Figure 7B). Thus, hydralazine likely counteracts the triggering mechanisms of GPMur/AE1-associated hypertension. Though it remains to be determined whether hydralazine could be more suitable for hypertensive patients with the GP.Mur blood type, this experiment did show applicability of the GPMur KI mouse model for antihypertensive research and development.^60,61^

**Figure 8.**
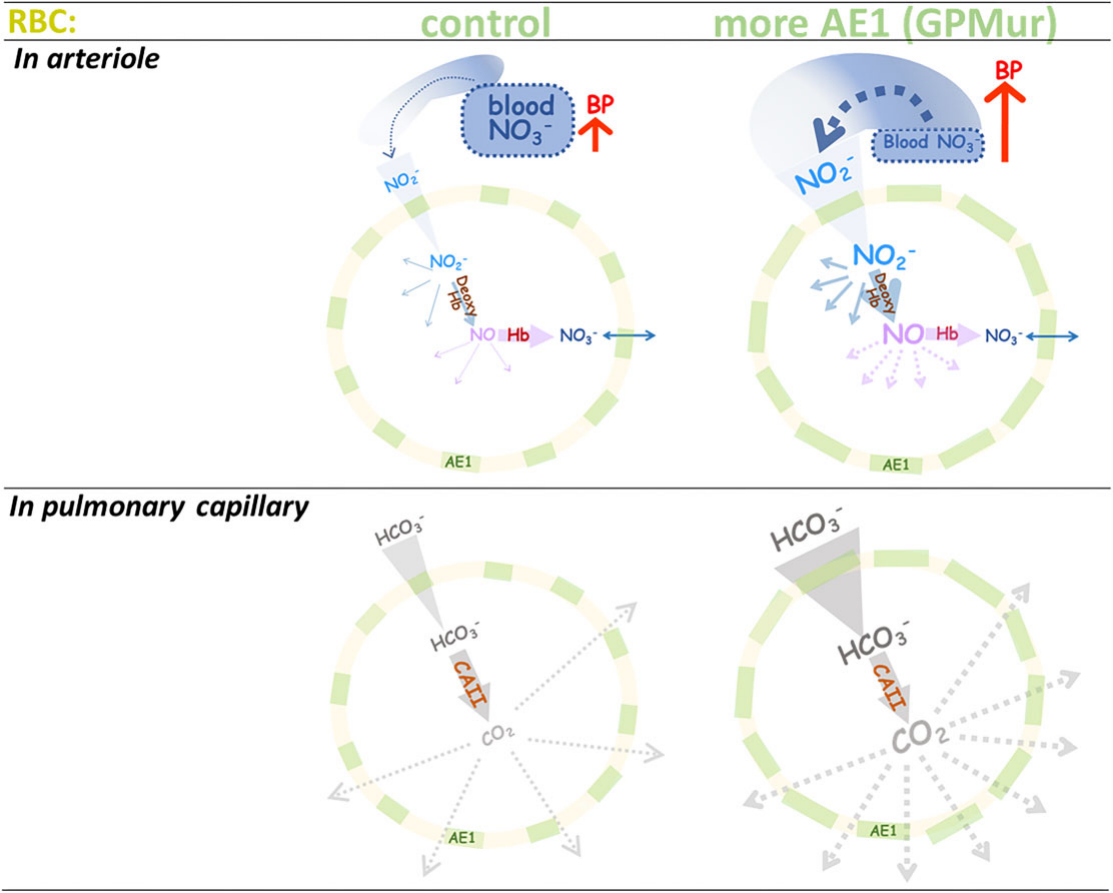
The dual roles of erythroid anion transport in NO and BP regulation and in CO_2_ respiration. (Top) From the comparative studies using the GPMur KI mice, the driving force for NO_2_^−^ influx/scavenging is stronger with a more anion-permeable erythroid membrane. NO_2_^−^_(aq)_ can be converted to NO_(g)_ and other forms of NO metabolites inside the RBCs. Nitric oxide gas inside erythrocytes is extremely rapidly converted to stable NO_3_^−^_(aq)_ by oxyHb. NO_3_^−^_(aq)_ can permeate in and out of the RBC through AE1. Lower NO_3_^−^_(aq)_ is observed in the bloodstream of GPMur KI mice. Reactive NO_2_^−^ that is constantly converted to even more reactive vasodilating NO and other NO metabolites may be supplied from NO_3_^−^ (the main storage form of NO_(g)_ in the body) by nitrate reductase in the microbiome (dotted arrows). GPMur/more AE1 RBCs scavenges NO_2_^−^ at faster rates and uses up NO_3_^−^ more rapidly, which may trigger higher BP. In the illustration, AE1 is symbolized as the gates or blocks on the RBC membrane; the blood NO_3_^−^ reservoir is symbolized in dotted boxes. (Bottom) AE1 on the RBC membrane transports HCO_3_^−^_(aq)_ bidirectionally, depending on the bicarbonate gradients across the cell membrane (which differ in different locations in the body). As RBCs circulate to the lung capillaries, where CO_2(g)_ pressure drops, blood HCO_3_^−^_(aq)_ rushes into the RBCs through AE1 to be converted to CO_2(g)_ for expiration by intraerythrocytic carbonic anhydrase II (CAII). The CO_2_ gas is not charged and can thus diffuse and leave the RBCs through lipid bilayer and transporters/channels.^76–79^ More AE1 on the RBC membrane (GPMur) accelerates influx of HCO_3_^−^_(aq)_ and conversion to CO_2(g)_ as RBCs circulate to the pulmonary capillaries. Note that the rate of erythroid anion transport via AE1 is much slower than the rates of the reactions facilitated by these intracellular enzymes, i.e. deoxyHb (top) and CAII (bottom), and thus AE1-mediated anion flux is the rate-limiting step in both physiologic phenomena.

Despite that our middle-aged GPMur KI mice showed ≥20 mmHg higher BP, they could run similarly well as the age-matched control mice on a rodent treadmill for over 1 h before reaching exhaustion [maximal endurance running distance: 1134.7 ± 232.2 m (5 month old GPMur) vs. 1073.3 ± 167.7 m (5 month old control); no significant differences]. Their serum NT proBNP (a marker elevated by ventricular pressure overload and myocardial overstretch) was also unremarkable and similar. Since these 2 groups of mice were on normal chow and were not stressed (eg, metabolically insulted by a high-fat diet), GP.Mur/AE1-associated hypertension conceivably could be considered “preclinical grade” and not yield cardiovascular complications in the absence of a stressor.

## Discussion

Here, we report the first hypertensive mouse model induced by the common SEA blood type GPMur. This murine GPMur/AE1 model demonstrates the impacts of overexpressed erythroid anion transporter AE1 on NO bioavailability and blood pressure.

How erythroid anion transporter is involved in Hb-NOx reactions and essential hypertension has long been unclear.^1–4^ Intraerythrocytic oxyHb is the predominant NO scavenger in the body that functions as a catalytic enzyme for the reaction: NO → NO_3_^−^. Different redox states of intraerythrocytic Hb also catalyze various conversion reactions between NO, NO_2_^−^, NO_3_^−^, and other NOx species.^53,54^ As the cytoplasmic domain of AE1 binds Hb (preferentially deoxyHb) in the inner leaflet of the erythrocyte membrane, it has been hypothesized that “the AE1-Hb metabolon” could “channel” fluxes of extracellular monoanionic NO metabolites (ie, NO_2_^−^ and NO_3_^−^) to submembranous Hb that catalyzes various NOx reactions.^7,62^ With the concept of “channeling” or “metabolon,” the energy cost for anion permeation across the cell membrane is much reduced for reactions near the perimembrane region (eg, CO_2_/HCO_3_^−^ conversion; nitrite reduction to NO inside the RBCs).^7,11^ We recently reported that intraerythrocytic NO_2_^−^/NO processing relies on AE1-mediated anion transport.^57^ Here, we used GPMur KI mice with higher AE1 expression, and found faster RBC scavenging of NO metabolites and higher BP in these animals (Figures 3-7 and Table 1).

In the KI mice, GPMur increases erythroid AE1 expression (Figures 2 and 3),^18^ and makes the murine RBC membrane more permeable to monoanions (ie, Cl^−^, HCO_3_^−^, NO_2_^−^, and NO_3_^−^) (Figure 8).^5,18^ Increased erythroid membrane permeation of HCO_3_^−^ through anion transporter AE1 accelerates CO_2_ excretion.^25,26^ Similarly, increased membrane permeation of NO_2_^−^ through AE1 accelerated intraerythrocytic NO_2_^−^/NO processing (Figures 5 and 6) and affected systemic NO_3_^−^ reservoir and blood pressure (Figure 4 and Table 1). These KI data support earlier findings in healthy people with GP.Mur—lower fractional exhaled NO (FeNO), lower FMD (rather due to stronger RBC scavenge of NO metabolites than endothelial defects), higher blood pressure, and stronger dependence of FMD and BP on individual Hb levels.^27–29^ Though both GPMur KI and eNOS knock-out present similar phenotypes—lower NO bioavailability and hypertension,^63^ their sites of action and mechanisms are different.

Since RBCs with GPMur/increased AE1 showed faster NO_2_^−^/NO processing (Figures 5 and 6), conceivably BP regulation in GPMur + mice/men is more sensitive to NO bioavailability.^45^ Indeed, hydralazine and amlodipine, both of which help maintain intravascular NO bioavailability, reduced BP more effectively in the GPMur mice (Figure 7). But their mechanisms for maintaining NO bioavailability are distinguishable.^64–68^ Amlodipine is pleiotropic as a CCB and a dual-action antioxidant that stimulates endothelial NO synthesis.^64,65^ Hydralazine is an old antihypertensive drug from the 1950s with complex mechanisms. Besides being a direct arterial vasodilator, hydralazine is a powerful scavenger of O_2_^•–^ and other ROS, which could suppress the formation of harmful ONOO^−^ from O_2_^•–^ and NO.^67,69^ By scavenging the other substrate (O_2_^•–^) that is required to form peroxynitrite with NO, hydralazine indirectly preserves systemic NO bioavailability. Nonetheless, our understanding of the pharmacologic mechanisms of hydralazine remain incomplete.^67,68,70^

In contrast, among the popular antihypertensive drugs tested, the effects of the angiotensin receptor blocker valsartan were similar for both mouse groups (Figure 7A), indicating that GPMur/increased AE1 did not affect the main mechanisms or sites of the action of the ARB.

Different from most murine hypertension models (eg, defects in the renin-angiotensin-aldosterone system [RAAS]; deletion of eNOS),^60,71,72^ KI of human *GYP.Mur* increased murine erythroid AE1 and blood pressure moderately without overkilling other BP-regulating physiological systems. Despite having lower systemic NO reservoir (Figure 4), CBC data (Table 1) and biochemical test results (Table 2) of these GPMur mice were all normal. We did not find “GPMur/AE1-triggered mild hypertension” to affect general health or longevity of the KI mice. This reflects that GP.Mur, as a common blood type among SEA is not lethal.^12,15–17^ On the other hand, in the GP.Mur-prevalent Eastern County of Taiwan (∼15% GP.Mur+, compared to ∼4.7% in overall Taiwan residents), the frequencies of stroke and early-onset hypertension appear slightly higher, according to annual reports from the Taiwan-National Health Insurance (TW-NHI). Conceivably, real-life stressors and various metabolic insults may exacerbate GPMur/AE1-associated hypertension risks.

From a public health survey on hypertension among different ethnic populations in New York City (NYC), East and SEAs and non-Hispanic blacks are ≥2.5 times more likely to have hypertension than non-Hispanic whites, after adjusted for age, sex, and education levels.^73^ Hispanic and South Asians in NYC are, respectively, 1.3 and 1.5 times more likely than non-Hispanic whites to have hypertension.^73^ As hypertension is a multifactorial illness influenced by genes, environment, and lifestyle/stress, the impacts of GP.Mur on hypertension risks, particularly on subtypes of hypertension and among different SEA populations, should be investigated in the future. Ethnic consideration in treating hypertension should not be overlooked. A well-known example is that ACE inhibitors and ARBs are not effective for treating hypertension in African Americans and African natives, as this ethnic group is generally low in renin.^74^ The establishment of the GPMur KI mouse model here could serve as a primer for future antihypertensive investigation, especially for the SEA populations.

### Limitation

A limitation of this study is the lack of BP telemetry, which is required for pharmaceutical development of antihypertensives. Blood pressure telemetry is technically challenging. Despite the lack of BP telemetry here, we adopted a conventional tail-cuff method for BP measurements and ensured that the mice were trained to be acquainted and comfortable with the procedure prior to the measurements (details described in the “Methods” section).

We found lower nitrate in hypertensive GPMur KI mice (Figure 4), though we have not tried to treat hypertension in these mice with a high-nitrate diet. We have only tested them with common antihypertensive drugs (Figure 7). This is because the effect of nitrate supplementation alone on the blood pressure of lab mouse remains controversial. Hezel et al. previously reported that nitrate supplementation in the diet of healthy middle- to old-aged lab mice could not improve their blood pressure.^75^ Conceivably, the study design may need to be optimized to show benefits of nitrate supplementation on mouse BP.

## Supplementary Material

zqae052_Supplemental_File

## Data Availability

The data underlying this article will be shared on reasonable request to the corresponding author.
