# Supplementary material for: Increased Anion Exchanger-1 (Band 3) on the Red Blood Cell Membrane Accelerates Scavenging of Nitric Oxide Metabolites and Predisposes Hypertension Risks
Source: Function (Oxf). 2024 Dec 4;6(1):zqae052. doi: 10.1093/function/zqae052 (PMC11815584; doi:10.1093/function/zqae052)

**Online Supplemental DATA**

**Supplemental Table 1. Complete Blood Count (CBC) tests for 4-month-old GPMur KI versus the control male mice.**

WBC, white blood cell; NEUT, neutrophil; LYMPH, lymphocyte; MONO, monocyte differential; EO, eosinophil differential; BASO, Basophil; HGB, hemoglobin; HCT, hematocrit; MCV, mean RBC corpuscular volume; MCH, mean RBC corpuscular hemoglobin content; MCHC, mean RBC corpuscular hemoglobin concentration; RDW, RBC corpuscular distribution width; RET, reticulocyte count; PLT platelet count; MPV-O, mean platelet volume; PCT, plateletcrit; PDW, platelet distribution width. *n.s.,* not significant.


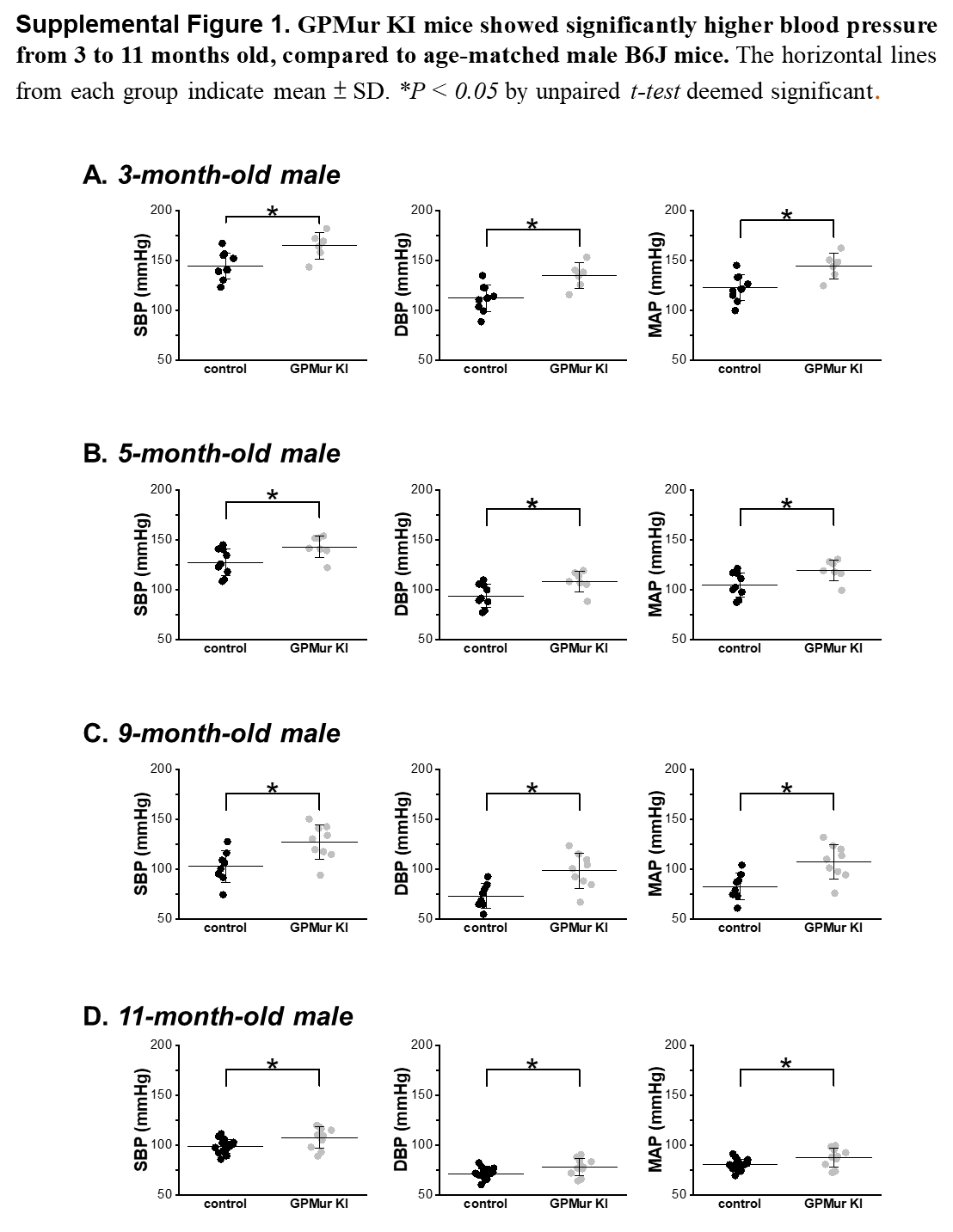

Supplement: zqae052_Supplemental_File [file zqae052_supplemental_file.docx]
